# The Effect of Cerium Oxide Nanoparticle Valence State on Reactive Oxygen Species and Toxicity

**DOI:** 10.1007/s12011-015-0297-4

**Published:** 2015-03-17

**Authors:** Katherine M. Dunnick, Rajalekshmi Pillai, Kelly L. Pisane, Aleksandr B. Stefaniak, Edward M. Sabolsky, Stephen S. Leonard

**Affiliations:** 1 National Institute for Occupational Safety and Health, HELD, 1095 Willowdale Rd, Morgantown, WV 26505 USA; 2 Pharmaceutical and Pharmacological Sciences, West Virginia University, Morgantown, WV 26505 USA; 3 Benjamin M. Statler College of Engineering and Mineral Resources, WVU, Morgantown, WV 26505 USA; 4 Department of Physics and Astronomy, West Virginia University, Morgantown, WV 26505 USA; 5 National Institute for Occupational Safety and Health, DRDS, Morgantown, WV 26505 USA

**Keywords:** Cerium oxide, Valence state, Nanotoxicology, Reactive oxygen species, Toxicity

## Abstract

Cerium oxide (CeO_2_) nanoparticles, which are used in a variety of products including solar cells, gas sensors, and catalysts, are expected to increase in industrial use. This will subsequently lead to additional occupational exposures, making toxicology screenings crucial. Previous toxicology studies have presented conflicting results as to the extent of CeO_2_ toxicity, which is hypothesized to be due to the ability of Ce to exist in both a +3 and +4 valence state. Thus, to study whether valence state and oxygen vacancy concentration are important in CeO_2_ toxicity, CeO_2_ nanoparticles were doped with gadolinium to adjust the cation (Ce, Gd) and anion (O) defect states. The hypothesis that doping would increase toxicity and decrease antioxidant abilities as a result of increased oxygen vacancies and inhibition of +3 to +4 transition was tested. Differences in toxicity and reactivity based on valence state were determined in RLE-6TN rat alveolar epithelial and NR8383 rat alveolar macrophage cells using enhanced dark field microscopy, electron paramagnetic resonance (EPR), and annexin V/propidium iodide cell viability stain. Results from EPR indicated that as doping increased, antioxidant potential decreased. Alternatively, doping had no effect on toxicity at 24 h. The present results imply that as doping increases, thus subsequently increasing the Ce^3+^/Ce^4+^ ratio, antioxidant potential decreases, suggesting that differences in reactivity of CeO_2_ are due to the ability of Ce to transition between the two valence states and the presence of increased oxygen vacancies, rather than dependent on a specific valence state.

**Electronic supplementary material:**

The online version of this article (doi:10.1007/s12011-015-0297-4) contains supplementary material, which is available to authorized users.

## Introduction

Cerium oxide (CeO_2_) nanoparticles are useful in a variety of applications, including polishing agents, solar cells, and catalysts; they have also found use as a diesel fuel additive [1, 2]. Cerium (Ce), a rare earth metal of the lanthanide series, is the most abundant rare earth metal making research into the production and use of CeO_2_ nanoparticles desirable. When in the form of CeO_2_, the Ce atom can exist in both a trivalent (Ce^3+^) and more stable tetravalent (Ce^4+^) state, allowing the nanoparticles to store and release oxygen [3]. This ability has increased industrial interest into CeO_2_ and its potential use in catalysts [4]. In fact, production of CeO_2_ with increased oxygen storage and releasing properties is desirable in industry to increase its catalytic properties. This increased interest will result in growth in the industrial uses of CeO_2_ and consequently result in greater exposure risks, specifically inhalation risks, for individuals working in the manufacturing process. Therefore, to understand and limit potentially toxic inhalation exposures, investigation into the toxicity of CeO_2_ is crucial.

Studies of the toxicity of this nanomaterial have been completed using various cell types, including pulmonary epithelial cells, macrophages, lung fibroblasts, and endothelial cells, but there have been conflicting results. For example, in pulmonary epithelial cells (BEAS-2B and A549 cells), CeO_2_ can either exert toxicity mediated by reactive oxygen species (ROS) production [5, 6] and Nrf-2 signaling [7] or has antioxidant-like properties [8]. Additionally, CeO_2_ has antioxidant-like properties under induced oxidative stress in RAW 264.7 macrophage cells [8] and protective effects against induced apoptosis in U937 and Jurkat lymphocyte cells [9]. These conflicting findings have been hypothesized to be a result of the ability of Ce to transition between Ce^3+^ and Ce^4+^ valence states and the subsequent oxygen vacancies formed from this transition [3, 10]. The reduction of Ce^4+^ to Ce^3+^ is thought to generate superoxide anions, which can produce damaging hydroxyl radicals. It is also postulated that Ce^3+^ can react with hydroxyl radicals and act as an antioxidant [6, 9–11]. Thus, it is possible that the valence state of Ce affects whether CeO_2_ nanoparticles play a protective or toxic role in exposed cells. Based on previous research, we hypothesize that valence state determines the extent of CeO_2_ toxicity and that when CeO_2_ exist in a greater 3+/4+ ratio, its toxicity will increase and antioxidant potential will decrease. To test this hypothesis and assess the effects of valence state, a technique known as doping was employed. Doping is the process of intentionally introducing impurities into a pure substance to modulate electrical properties. To modulate the oxygen storage and release capacity of CeO_2_ nanoparticles, rare earth metal ions with low valence states are typically used [4]. For this study, gadolinium(III) oxide (Gd_2_O_3_) was used to produce increased oxygen vacancies in the CeO_2_ nanoparticle lattice [4] and force the valence state toward a greater +3/+4 ratio. Two types of doped CeO_2_ nanoparticles were prepared and used for this study, a 10 and 20 mol% Gd in CeO_2_. In addition, pure CeO_2_ nanoparticles were tested. Previous studies have shown that Gd_2_O_3_ itself exhibits toxicity [12]; therefore, Gd_2_O_3_ controls were used throughout the study to ensure any differing effects between cerium compounds were due to valence state and transitional ability rather than the presence of Gd_2_O_3_. The effect of valence state and transitional ability of pure CeO_2_ nanoparticles and doped CeO_2_ nanoparticles on ROS and toxicity was assessed.

## Materials and Methods

### Cell Culture

RLE-6TN rat alveolar type II cells (ATCC; Rockville, MD) were cultured following a modified ATCC recommended protocol. Cells were cultured in Ham’s F12 medium with 5 % fetal bovine serum and 50 mg/ml penicillin/streptomycin (Thermo Scientific; Pittsburgh, PA). Cells were grown at 37 °C in a 5 % CO_2_ incubator and were passaged following trypsinization. RLE-6TN cells were chosen for these studies to represent the pulmonary alveolar region most likely to come into contact with nanoparticles. NR8383 rat macrophage cells (ATCC; Rockville, MD) were cultured following the ATCC recommended protocol. Cells were cultured in Ham’s F12K medium with 15 % fetal bovine serum and 50 mg/ml penicillin/streptomycin. Cells were grown at 37 °C in a 5 % CO_2_ incubator and were passaged by transferring floating cells to culture flasks.

### CeO_2_ Nanoparticle Production and Characterization

**Fig. 1:**
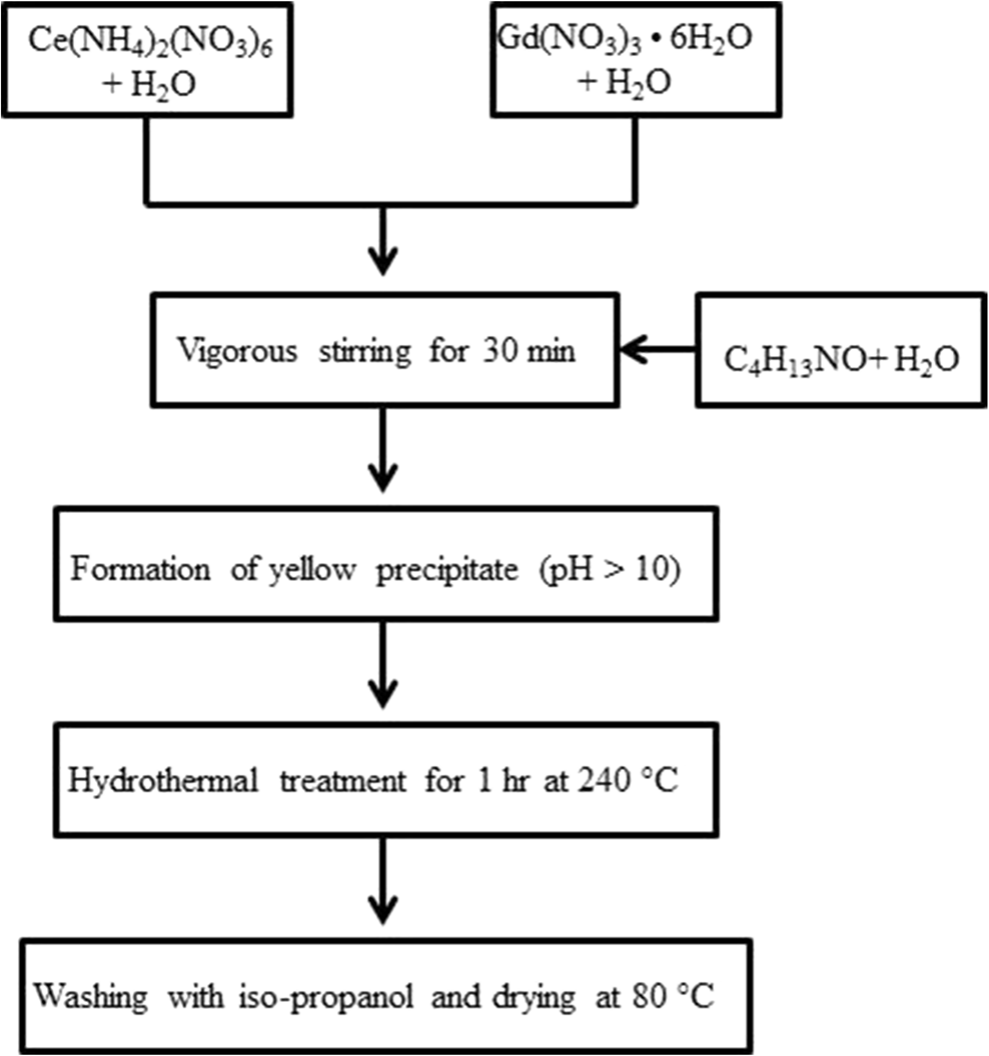
Synthesis of CeO_2_ nanoparticles by a hydrothermal method

Gd-doped CeO_2_ nanopowder was prepared using a hydrothermal method [13] (Fig. 1). For this process, two separate aqueous solutions (5 × 10^−3^ mol L^−1^) of cerium (IV) ammonium nitrate (Ce(NH_4_)_2_(NO_3_)_6_, 99.9 % purity), and gadolinium nitrate hexa-hydrate (Gd(NO_3_)_3_·6H_2_O) were prepared by dissolving the salts into deionized water at room temperature. The as-prepared solutions were mixed together under vigorous stirring. An aqueous solution of tetramethyl ammonium hydroxide (TMAH) was added drop by drop until the pH of the solution reached 10. After 30 min of stirring, a white or yellowish gel-like precipitate was formed and settled rapidly. The supernatant of the solution was decanted, and the resulting solid was rinsed several times with deionized water and hydrothermally treated at 240 °C for 1 h under autogenous pressure without stirring to obtain cerium (or Gd-doped cerium) oxide. The clear supernatant was decanted, and the yellowish precipitate was washed with isopropanol and then dried at 80–85 °C overnight.

An X’PERT PRO Panalytical X-ray diffractometer (Westborough, MA) was used to determine the phase of the prepared ceria powders using Cu Kα radiation. Data was collected from 10°–90° angles (2θ) with a step size of 0.02 increments at a rate of 1°/min. Phase identification was achieved by X’PERT PRO software through the comparison of indexed powder diffraction files maintained by International Center for Diffraction Data. The morphology of the synthesized ceria powders was examined by scanning electron microscopy (SEM; JEOL 7600F; Peabody, MA). Energy-dispersive X-ray spectroscopy (EDS) was used to identify the elemental composition of the prepared powders. The X-ray photoelectron spectroscopy (XPS) measurements were carried out using a Physical Electronics, PHI 5000 Versa Probe (XPS/UPS) spectrometer (Chanhassen, MN) with a monochromatic Al Kα source operated at 300 W and a base pressure of 5 × 10^−8^ Torr. XPS is a surface-sensitive technique that analyzes the top 25 to 50 angstroms of a particles exterior. The spectrometer was configured to operate at high resolution with energy of 100 eV. The acquisition time of the sample was kept low to minimize surface oxidation state changes during X-ray irradiation. The XPS analysis was performed to understand the changes in the valence state and binding energy of the constituent elements on powder surfaces. The work function of the instrument was calibrated to a binding energy of 83.96 eV for the Au 4f7/2 line for metallic gold, and the dispersion of the spectrometer was adjusted to a binding energy of 932.62 eV. The powder samples were placed on the sample holder using a double-sided conductive tape followed by 6-h evacuation prior to analyses. Survey spectra were collected by 1.0-eV steps at analyzer pass energy of 160 eV and the high-resolution analysis of small spectrum regions by 0.05-eV steps and pass energy of 20 eV. The integrated area under the curve of each de-convoluted peak was used to calculate the concentration of Ce^3+^ ions as $\left[Ce\right]=\frac{\left[A{v}_0+A{v}^{\prime }+A{u}_0+A{u}^{\prime}\right]}{{\displaystyle \sum {A}_i}}$ where *A*_*i*_ is the integrated area for peak *i*.

The size distributions of CeO_2_ and Gd-doped CeO_2_ nanoparticles in a suspended state were assessed using dynamic light scattering (DLS). DLS analyzes the velocity distribution of suspended particles by detecting fluctuations of scattered light produced by Brownian motion of the particles and provides hydrodynamic radius or diameter of the particles. All measurements were performed using a Nano ZS90 instrument (Malvern Instruments; Worcestershire, UK). Prior to measurement, each sample cell was cleaned, rinsed with 0.02-μm filtered water, and pre-wetted with dispersion media (DM). Suspensions of each material in DM were subjected to ultrasonic agitation using a probe tip for 10 to 20 min (delivered energy = 4500 to 9000 J) until a uniform dispersion appeared. An ice bath was used to cool the samples during sonication.

The zeta potentials of CeO_2_ and Gd-doped CeO_2_ nanoparticles in a suspended state were assessed to describe the stability of the dispersions in DM. All measurements were performed using a Nano ZS90 instrument (Malvern Instruments; Worcestershire, UK). Prior to measurement, each sample cell was cleaned and rinsed with 0.02-μm filtered water and ethanol. All dispersant media were filtered through a 0.02-μm membrane prior to use as well. The viscosity of the dispersant was determined at room temperature using a VS-10 viscometer (Malvern Instruments), and measured values were used in the calculation of zeta potential. Each nanoparticle suspension was subjected to ultrasonic agitation for up to 10 min using a probe tip (delivered energy = 4400 J). The Smoluchowski approximation of 1.5 was used for Henry’s function, and a pH of 7.51 was determined for the DM.

Nitrogen gas adsorption was used to determine powder-specific surface area (SSA) using a multipoint Brunauer, Emmett, and Teller (BET) instrument (ASAP2020 surface area analyzer; Micromeritics; Norcross, GA). Prior to analysis, powders were outgassed under vacuum (0.013 Torr) for 3 h at 150 °C to remove moisture [14]. The transmission electron microscopy (TEM) samples were prepared by sonicating a mixture of CeO_2_ nanopowder and DM for 2 min to disperse the nanoparticles. Ethanol was added and the solution was sonicated for an additional 5 min. One drop of the resulting solution was placed on a carbon-coated copper TEM grid for imaging on a JEOL JEM 2100 (Peabody, MA) TEM with a LaB_6_ filament operated at 200 kV. Regular micrographs were taken with a Gatan ES500W (Gatan; Pleasanton, CA) digital camera, and high-resolution images were obtained with an Orius SC1000 (Gatan; Pleasanton, CA) camera.

### Determination of Cellular Interaction

To visualize nanoparticles, which are not visible using typical light microscopy, enhanced darkfield microscopy was employed [15]. RLE-6TN and NR8383 cells were grown on cleaned, autoclaved cover-glass (Chemglass Life Sciences; Vineland, NJ) until 60–80 % confluent. CeO_2_, Gd-doped CeO_2_, and Gd_2_O_3_ nanoparticles were prepared in DM at a stock concentration of 1 mg/ml, as previously described [16]. Cells were then treated with CeO_2_ or Gd_2_O_3_ (Sigma-Aldrich; St. Louis, MO) nanoparticles at a final concentration of 10 μg/ml for 5 min, 1 h, and 3 h. Following incubation, the medium was removed and the cells were washed three times with warm phosphate-buffered saline (PBS), fixed with 10 % formalin for 10 min, washed three times with PBS, mounted with Fluoromount G (eBioscience; San Diego, CA), and sealed with clear nail polish. Slides used for this experiment were purchased as clean cut slides to avoid silica particle residue, which results in high background during imaging (Schott Nexterion, Arlington, VA). Following mounting, images were acquired at 60x magnification using a Cytoviva enhanced darkfield microscopy system (Aetos Technologies; Inc., Auburn, AL) integrated into an Olympus BX41 upright optical microscope equipped with an Olympus DP73 digital camera (Olympus; Center Valley, PA).

### Electron Paramagnetic Resonance (EPR)

A spin trap technique was used to form long-lived free radicals that could be detected by EPR through addition of 5-(diethioxyphosphoyl)-5-methyl-1-pyrroline *N*-oxide (DEPMPO) or 5,5′-dimethylpyrroline *N*-oxide (DMPO). EPR measurements were collected using a flat cell assembly and Brüker EMX spectrometer (Billerica, MA). CeO_2_ and Gd-doped CeO_2_ nanoparticles were incubated at a final concentration of 1 mg/ml with 50 mM DEPMPO (Cayman Chemical, Ann Arbor, Michigan), 3.5 mM xanthine, and 2 U/ml xanthine oxidase (Sigma-Aldrich) for 3 min to produce superoxide radicals. To induce hydroxyl radicals in an acellular system and assess antioxidant potential, CeO_2_ and Gd-doped CeO_2_ were incubated at a final concentration of 1 mg/ml with 100 mM DMPO (Sigma-Aldrich) and 1 mM H_2_O_2_ and then exposed to UV light for 1 min. The mass of Gd_2_O_3_ powder was adjusted to achieve a final concentration of 179 μg/ml, as this value represents the theoretical amount of elemental Gd in the 20 mol% Gd-doped CeO_2_ nanoparticles. This reaction was also run in the absence of UV light to assess the ability of CeO_2_ and Gd-doped CeO_2_ to produce hydroxyl radicals. Samples were run in triplicate, and instrument settings are indicated under “Results.” Signal intensity (peak height) was used to measure the relative amount of superoxide radicals produced and is measured in millimeters.

For cellular EPR, CeO_2_ and Gd-doped CeO_2_ at final concentrations of 1 mg/ml or Gd_2_O_3_ at 179 μg/ml were incubated with either RLE-6TN or NR8383 cells at 2 × 10^6^ cells/ml and 200 mM DMPO for 3 min at 37 °C [17, 18]. Reactions were run in triplicate. This reaction was repeated but 2 mM Cr(VI) was added to the system to induce hydroxyl radicals. Peak heights represent relative amounts of hydroxyl radicals produced and are measured in millimeters.

### Annexin V/Propidium Iodide

The degree of apoptosis and necrosis induced by CeO_2_ and Gd-doped CeO_2_ at 24 h was determined by flow cytometry. RLE-6TN cells were seeded at 1 × 10^5^ cells per well in 24-well plates, and NR8383 cells were seeded at 3 × 10^5^ cells per well. Following 24 h of growth, cells were treated with CeO_2_ and Gd-doped CeO_2_ at a final concentration of 10 or 50 μg/ml for 24 h or treated with Gd_2_O_3_ at a final concentration of 1.79 or 8.95 μg/ml. The annexin V/propidium iodide assay was completed according to company protocol (Trevigen; Gaithersburg, MD). Briefly, cell media were collected followed by trypsinization of cells for 2 min. Trypsinized cells were combined with media to ensure collection of viable, apoptotic, and necrotic cells. Following a washing step, cells were incubated for 15 min with 100 μl annexin V/propidium iodide stain then analyzed on a BD Biosciences LSR II flow cytometer. All data were analyzed using DIVA software and 10,000 events per sample were collected. Samples were run three times in duplicate and are presented in graphical rather than scatter plot format.

### Statistical Analysis

All data are represented as the mean ± standard deviation for each condition. To compare responses between groups, a one-way analysis of variance (ANOVA) and Tukey posttest were performed using GraphPad Prism 6 software (GraphPad Software, Inc.; La Jolla, CA). Statistical significance is shown when *p* < 0.05.

## Results

### CeO_2_ Characteristics

The XRD diffraction peaks of the CeO_2_, which represent the crystalline plane (1 1 1), (2 0 0), (2 2 0), and (3 1 1), correspond to cubic fluorite crystal structure (JCPDS Data Card # 88-2326), where Ce is in the 4+ oxidation state [19]. The XRD pattern of CeO_2_ 10 % Gd and CeO_2_ 20 % Gd showed no Gd oxide peaks, indicating the formation of Gd-CeO_2_ solid solution [19] (data not shown). SEM was used to assess the agglomeration of the nanoparticles (Online Resource 1) and indicated that the CeO_2_ and doped-CeO_2_ powders agglomerated and that there was a wide distribution of particle sizes. The EDS pattern of pure CeO_2_ (data not shown) did not reveal any impurities present in the powder.

**Fig. 2:**
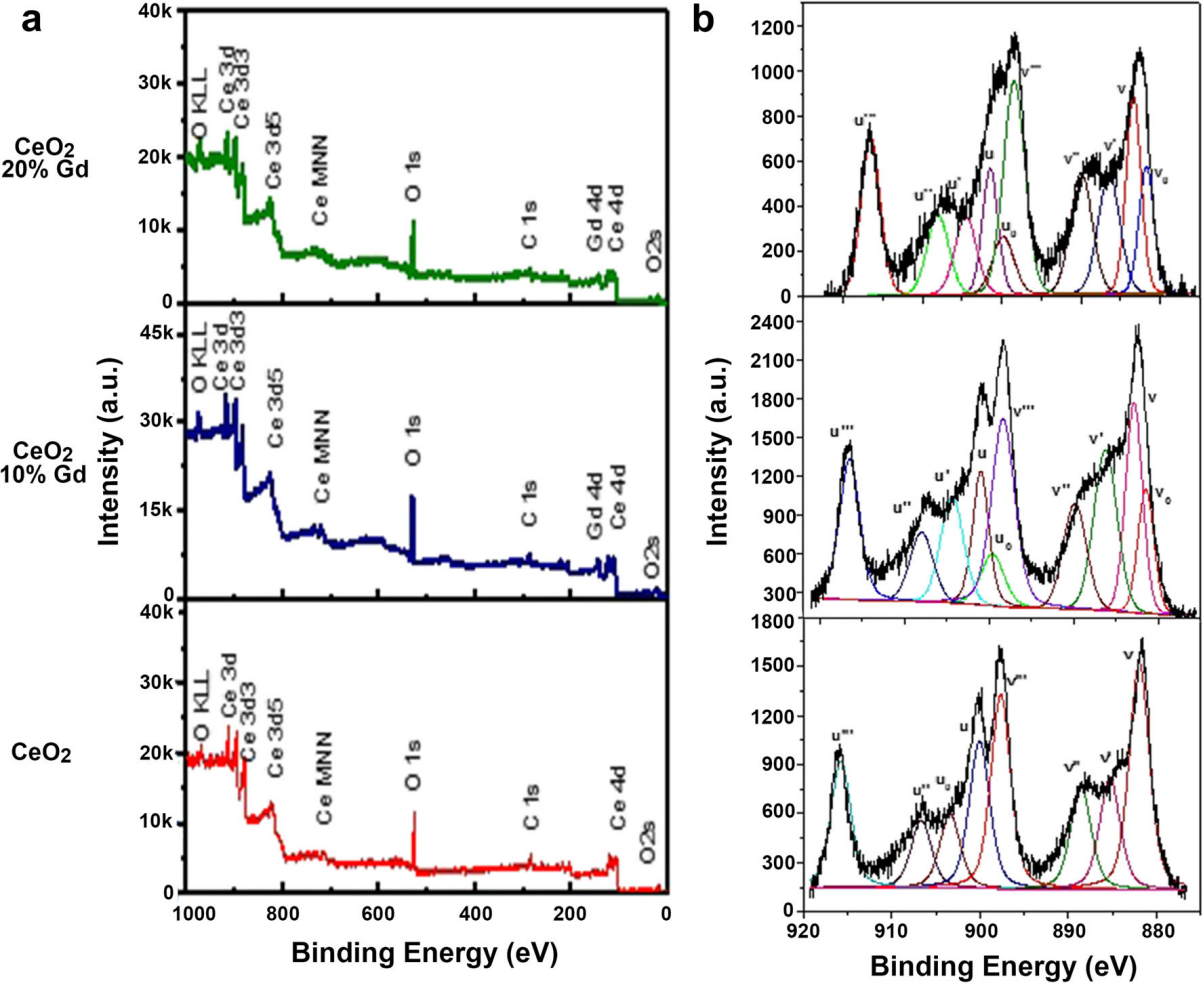
XPS survey of CeO_2_ nanoparticles. **a** Wide-scan XPS survey scan spectrum of CeO_2_ 20 % Gd, CeO_2_ 10 % Gd, and pure CeO_2_. **b** High-resolution XPS spectrum of CeO_2_ 20 % Gd, CeO_2_ 10 % Gd, and pure CeO_2_

Figure 2a shows the wide scan XPS survey spectra for pure CeO_2_, CeO_2_ 10 % Gd, and CeO_2_ 20 % Gd. High-resolution XPS spectra for Ce (3d), the fitted curve, and the corresponding de-convoluted peaks of CeO_2_ nanoparticles are shown in Fig. 2b. The recorded XPS spectra were charge corrected with respect to the C (1s) peak at 284.6 eV. The peaks in the spectrum of Ce were de-convoluted using the multi-pack software. The letter “v” marked in the spectra indicates the spin-orbit coupling 3d_5/2_, and the letter “u” indicates spin orbit coupling 3d_3/2_ of pure CeO_2_. The peaks denoted by *v*_0_, *v*′, *u*_0_, and *u*′ represent Ce^3+^ ions, whereas those marked by *v*, *v*″, *v*′′′, *u*, *u*″, and *u*′′′ represent Ce^4+^ ions. It is evident that the de-convoluted Ce (3d) spectrum is relatively complex due to the presence of Ce in 3+ and 4+ oxidation states as well as multiple d-splitting. The spin orbit doublets for pure CeO_2_, 3d_3/2_ (885.3 and 903.4 eV), and 3d_5/2_ (881.9 and 888.6 eV) are clearly evident for both valence states of Ce, indicating that Ce is in mixed valence states of 3+ and 4+ [20]. High-resolution XPS spectra for Ce (3d), the fitted curve, and the corresponding de-convoluted peaks of pure CeO_2_, CeO_2_ 10 % Gd, and CeO_2_ 20 % Gd are presented in Fig. 2. Table 1 shows the binding energies, peak heights, peak areas, and the concentrations of Ce^3+^ and Ce^4+^ atoms of pure CeO_2_, CeO_2_ 10 % Gd, and CeO_2_ 20 % Gd. The characteristic peaks of Gd^3+^ 3d_5/2_ were observed in the region 1183.83 ± 0.7 and 1215.83 ± 0.7 eV in CeO_2_ 10 % Gd and 1187.07 ± 0.7 and 1219.07 ± 0.7 eV in CeO_2_ 20 % Gd, indicating that Gd is in the 3+ oxidation state (Fig. 2). It was observed that in both the peaks of Gd^3+^, there was a slight shift toward the lower binding energy, which can be attributed to the increase in valence electron density. From the table, it may be seen that the addition of Gd increases the Ce^3+^ state. The ratios of Ce^3+^/Ce^4+^ were found to be 16.9, 42.7, and 43.9 % for pure CeO_2_, CeO_2_ 10 % Gd, and CeO_2_ 20 % Gd, respectively. The high value of *v*_0_/*u*_0_ and *v*′/*u*′ indicates that nanosized ceria exhibits better catalytic activity due to the large amount of electronic and ionic defects, which include the presence of Ce^3+^ and Gd^3+^ atoms and the corresponding oxygen vacancies (*V*_O_^··^). Gd is a lanthanide that can be used to modify the chemical, crystal structure, and defect state of ceria. The atomic radius and the electron negativity of Gd are close to that of the cerium atom, so the atom fits into the Ce-site within the fluorite structure. It must be noted that, as the amount of Ce^3+^ and Gd^3+^ states within the structure increases, the structure must compensate for these additions by increasing the positive charge within the material to retain charge neutrality. The material typically compensates for this ionic defect by releasing oxygen from the structure, resulting in an open anionic site within the structure (oxygen vacancy, V_O_^··^). The oxygen vacancies may be considered as open sites within the bulk and surface structure for the uptake of oxygen and are critical for the efficient diffusion of oxygen ions within or on the surface of the ceria.

**Table 1:** XPS analysis of Ce^3+^ and Ce^4+^ ion concentration

Samples	Binding energy (eV)	Peak height	Peak area		[Ce^4+^]	[Ce^3+^]	Ce^3+^/Ce^4+^
CeO_2_	881.96	1382	4717	Ce^4+^	13,981	2363	0.169
	885.3	683	2363	Ce^3+^			
	888.57	625	2166	Ce^4+^			
	897.66	1184	3985	Ce^4+^			
	900.06	899	3113	Ce^4+^			
CeO_2_ 10 % Gd	881.49	781	1944	Ce^3+^	13,790	5892	0.427
	883.02	1512	4306	Ce^4+^			
	886.13	1096	3948	Ce^3+^			
	889.55	838	3134	Ce^4+^			
	898.42	1616	6350	Ce^4+^			
CeO_2_ 20 % Gd	881.6	563	1401	Ce^3+^	7525	3301	0.439
	883.33	876	2181	Ce^4+^			
	886.42	527	1900	Ce^3+^			
	889.92	538	1938	Ce^4+^			
	898.4	946	3406	Ce^4+^			

Hydrodynamic diameter and zeta potential were measured to assess particle agglomeration under physiological exposure conditions, while TEM was used to observe particle size. The results indicated that the hydrodynamic diameters of all three CeO_2_ nanoparticles (CeO_2_, 875 ± 58; CeO_2_ 10 % Gd, 201 ± 5; CeO_2_ 20 % Gd, 176 ± 8) (Table 2) were larger than the observed size under TEM (~5 nm) (Online Resource 2). The zeta potential indicates that the nanoparticle dispersions are likely to agglomerate in DM (Table 2) based on the stability categories developed by Riddick [21]. Thus, the results show that the stability of the nanoparticle dispersions is fairly poor overtime. The surface area results implicate that the pure CeO_2_ and CeO_2_ 10 % Gd were of similar surface area, while the surface area of CeO_2_ 20 % Gd was substantially less (Table 2).

**Table 2:** Characteristics of pure and doped CeO_2_ nanoparticles

Nanoparticle	Hydrodynamic diameter (nm)	Zeta potential	Surface area (CV %)
CeO_2_	875 ± 58	−10.6 ± 2.4	204.8 ± 14.6 (7.1)
CeO_2_ 10 % Gd	201 ± 5	−16.3 ± 2.6	225.4 ± 34.1 (15.1)
CeO_2_ 20 % Gd	176 ± 8	−12.8 ± 1.6	135.6 ± 5.6 (4.1)

### Cellular Interactions with Particles Show Accumulation Over Time

**Fig. 3:**
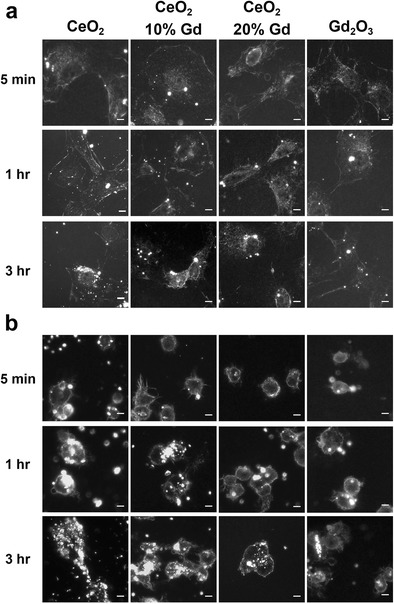
Epithelial and macrophage cells associate with CeO_2_ nanoparticles over a time course. **a** Cytoviva-enhanced dark-field microscopy system provides images of high-contrast CeO_2_ nanoparticles (*bright spots*) against a *dark background* of cells. Cells were exposed to CeO_2_ nanoparticles for 5 min, 1 h, or 3 h. **a** Representative images of RLE-6TN cells associated with CeO_2_ nanoparticles. **b** As in **a**, except images are representative of NR8383 cells. *Scale bar*, 5 μm

Enhanced darkfield microscopy was used to visualize CeO_2_ and Gd_2_O_3_ nanoparticle cellular interactions over a time course of 3 h. The results demonstrated that all CeO_2_ nanoparticles and Gd_2_O_3_ accumulated with cells over time (Fig. 3). Figure 3b illustrates that all nanoparticles associated with NR8383 cells more rapidly than RLE-6TN cells.

### Super Oxide Radical Scavenging with CeO_2_ Nanoparticles

**Fig. 4:**
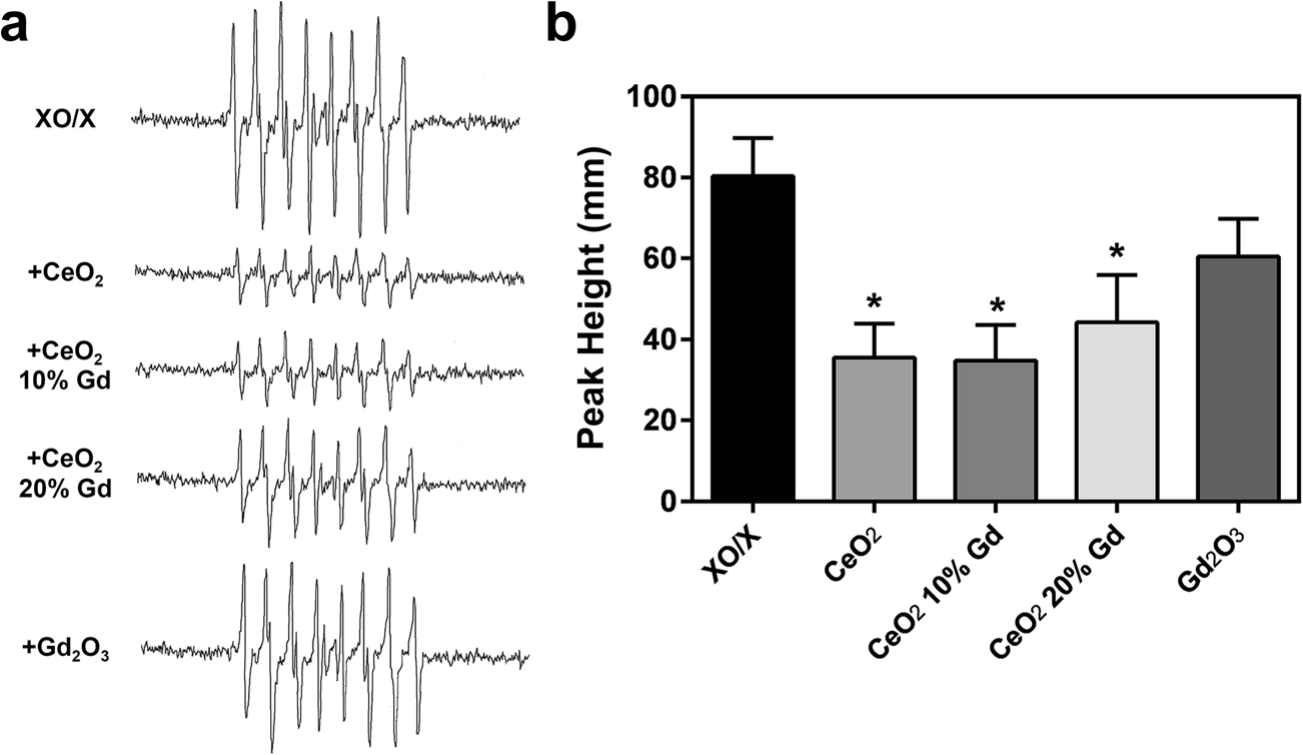
CeO_2_ nanoparticles reduce superoxide radicals. **a** CeO_2_ nanoparticles at 1 mg/ml (Gd_2_O_3_ at 179 μg/ml) were combined with 50 mM DEPMPO, 3.5 mM xanthine, and 2 U/ml xanthine oxidase (XO/X) for 3 min. EPR setting were the following: center field, 3490 G; scan width, 200 G; time constant, 0.41 s; modulation amplitude, 1 G; receiver gain, 2.5 × 10^4^; frequency, 9.8 GHz; and power, 63 mW. Representative spectra for each sample are shown. **b** The first, fourth, fifth, and eighth peaks were used for measurement of superoxide radical production. Signal intensity was measured in millimeters. *Error bars* represent the mean ± standard deviation. *p < 0.05 compared to XO/X

Studies have indicated that CeO_2_ has superoxide dismutase properties [11]; thus, the effect of doping and alteration in valence state on superoxide scavenging was assessed using a xanthine oxidase/xanthine reaction and spin trap technique. Results showed that all three CeO_2_ nanoparticles had significant scavenging properties in a 3-min acellular system; however, the Gd_2_O_3_ positive control did not have this effect (Fig. 4).

### Hydroxyl Radical Scavenging with CeO_2_ Nanoparticles

**Fig. 5:**
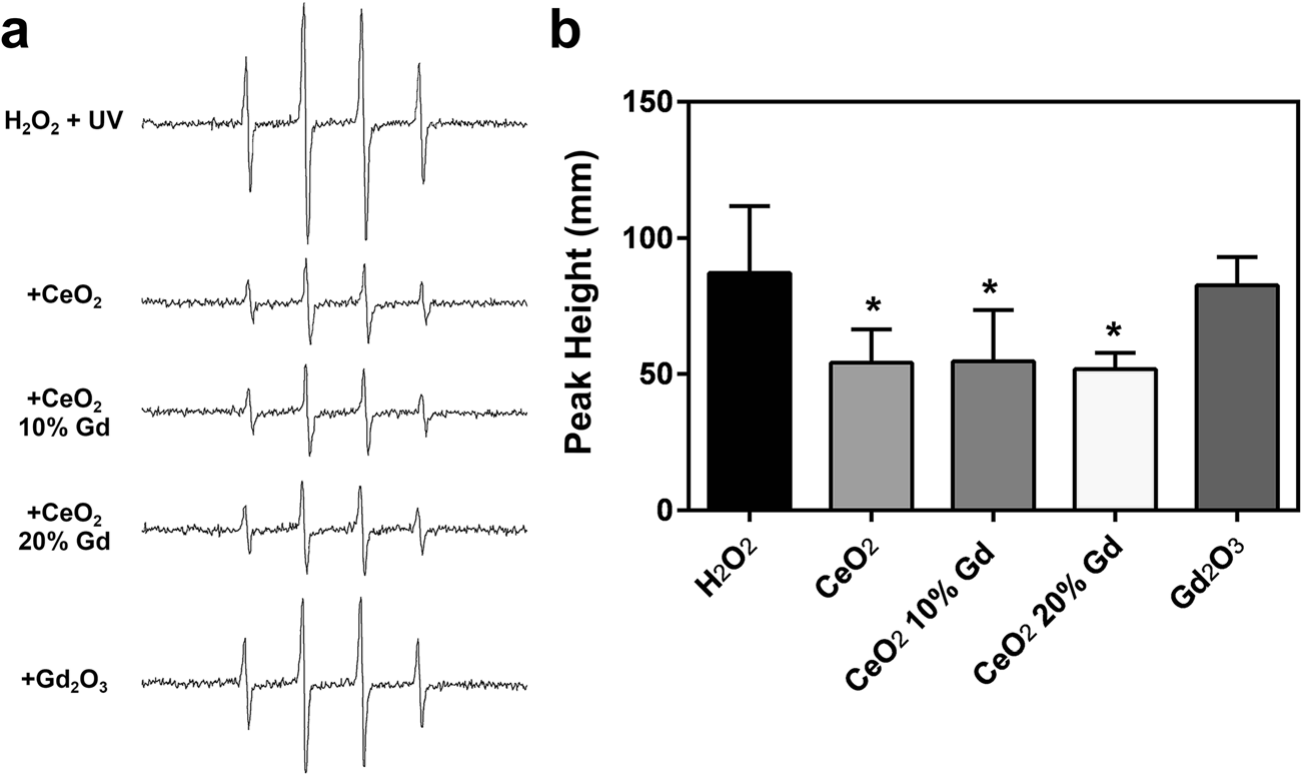
CeO_2_ nanoparticles reduce hydroxyl radicals. **a** CeO_2_ nanoparticles at 1 mg/ml (Gd_2_O_3_ at 179 μg/ml) were combined with 100 mM DMPO and 1 mM H_2_O_2_ then exposed to UV light for 1 min. EPR setting were the following: center field, 3487 G; scan width, 100 G; time constant, 0.41 s; modulation amplitude, 1 G; receiver gain, 2.5 × 10^4^; frequency, 9.8 GHz; and power, 63 mW. Representative spectra for each sample are shown. **b** The second and third peaks were used for measurement of hydroxyl radical production. Signal intensity was measured in millimeters. *Error bars* represent the mean ± standard deviation. *p < 0.05 compared to H_2_O_2_

As a result of the rapid association of nanoparticles with cells (within 5 min) and previous studies implicating that CeO_2_ can induce or scavenge ROS [7, 11, 22], hydroxyl radical production was measured. To determine whether CeO_2_ and Gd_2_O_3_ nanoparticles are capable of converting H_2_O_2_ into hydroxyl radicals, acellular Fenton-like reactions were carried out using EPR and a spin trap method. Neither CeO_2_ (pure and doped) nor Gd_2_O_3_ induced hydroxyl radicals in an acellular system (data not shown). Further, because previous studies have shown that CeO_2_ has scavenging abilities [8], the ability of CeO_2_ to scavenge hydroxyl radicals was carried out using H_2_O_2_, UV light, and a spin trap method. Results indicated that pure CeO_2_, CeO_2_ 10 % Gd, and CeO_2_ 20 % Gd had significant antioxidant effects, while Gd_2_O_3_ had no significant effects on induced hydroxyl radicals within 3 min in an acellular system (Fig. 5).

**Fig. 6:**
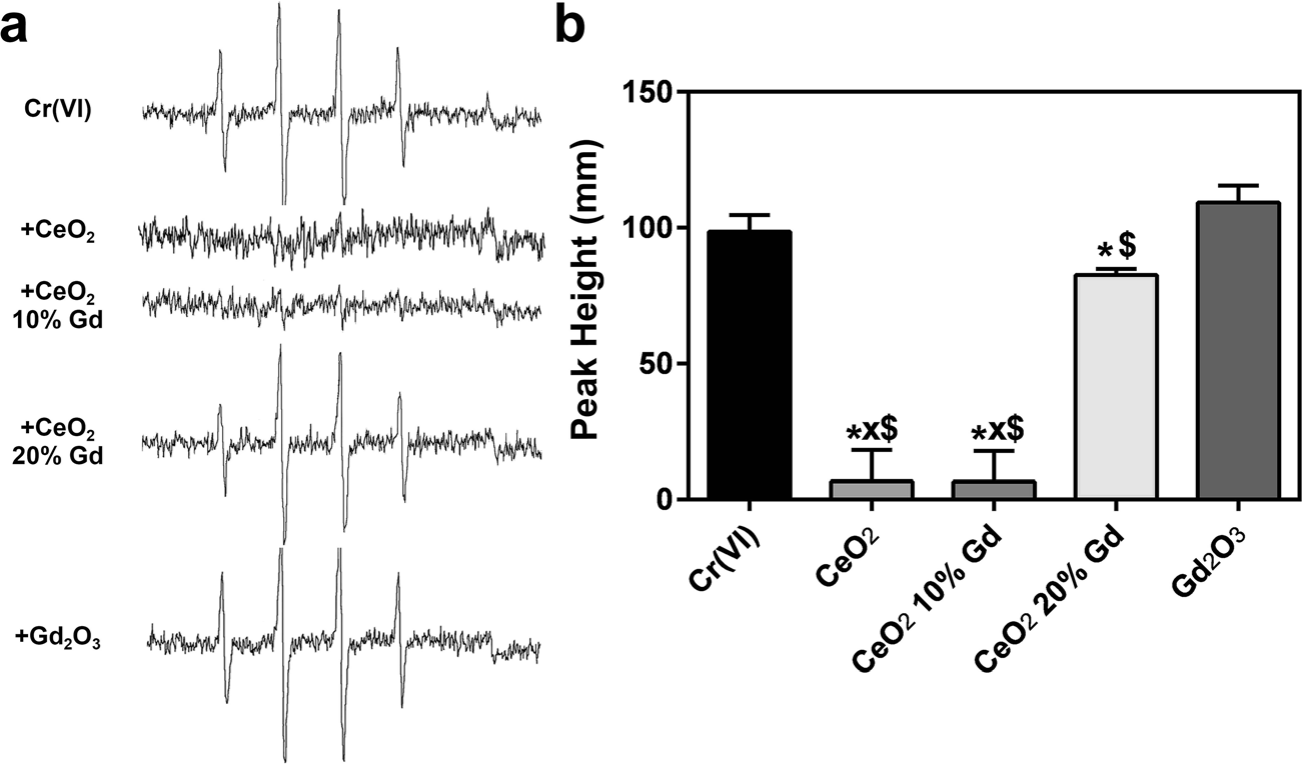
CeO_2_ nanoparticles reduce induced hydroxyl radicals in RLE-6TN cells. **a** CeO_2_ nanoparticles at 1 mg/ml (Gd_2_O_3_ at 179 μg/ml) were combined with 200 mM DMPO and 2 × 10^6^ cells/ml then incubated for 3 min at 37 °C. EPR setting were the following: center field, 3495 G; scan width, 100 G; time constant, 0.41 s; modulation amplitude, 1 G; receiver gain, 6.3 × 10^2^; frequency, 9.8 GHz; and power, 126 mW. Representative spectra for each sample are shown. **b** The second and third peaks were used for measurement of hydroxyl radical production. Signal intensity was measured in millimeters. *Error bars* represent the mean ± standard deviation. *p < 0.05 compared to control, *xp* < 0.05 compared to CeO_2_ 20 % Gd, $*p* < 0.05 compared to Gd_2_O_3_

**Fig. 7:**
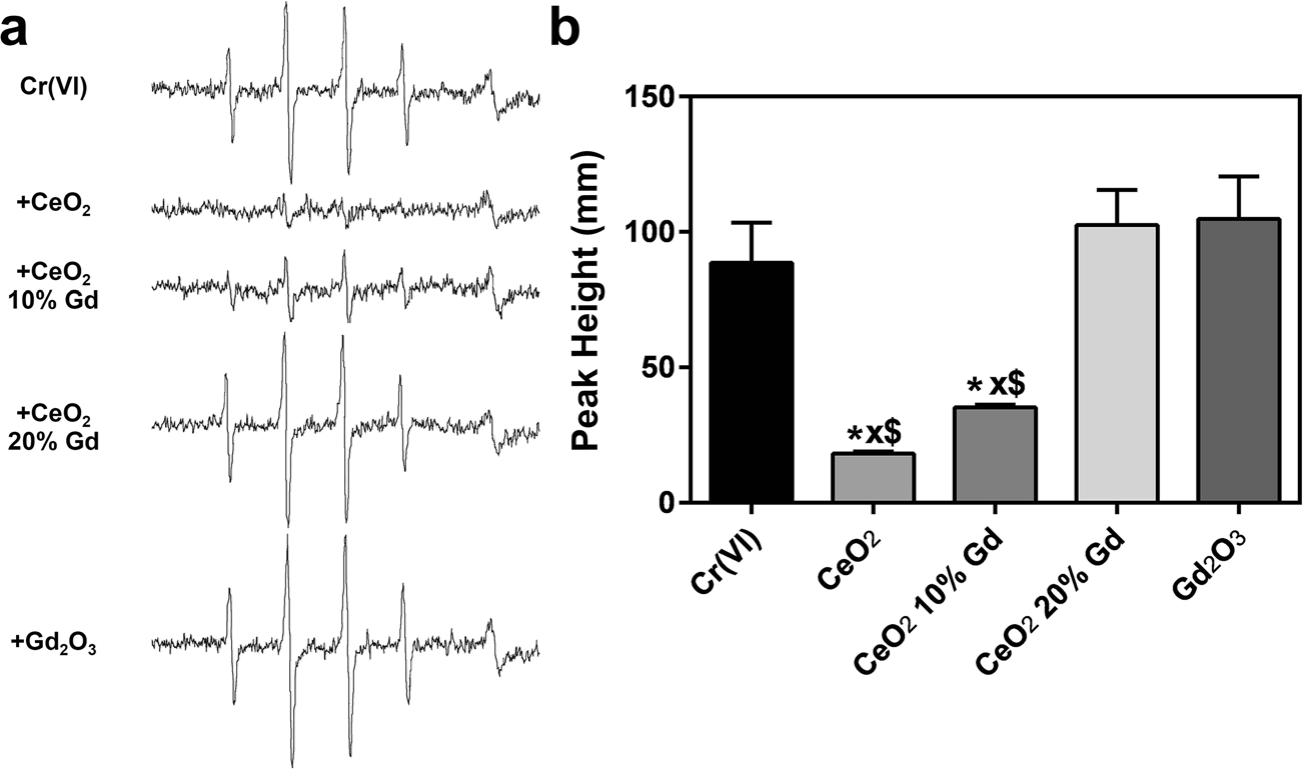
CeO_2_ nanoparticles reduce induced hydroxyl radicals in NR8383 cells. **a** CeO_2_ nanoparticles at 1 mg/ml (Gd_2_O_3_ at 179 μg/ml) were combined with 200 mM DMPO and 2 × 10^6^ cells/ml then incubated for 3 min at 37 °C. EPR setting were the following: center field, 3495 G; scan width, 100 G; time constant, 0.41 s; modulation amplitude, 1 G; receiver gain, 6.3 × 10^2^; frequency, 9.8 GHz; and power, 126 mW. Representative spectra for each sample are shown. **b** The second and third peaks were used for measurement of hydroxyl radical production. Signal intensity was measured in millimeters. *Error bars* represent the mean ± standard deviation. *p < 0.05 compared to control, *xp* < 0.05 compared to CeO_2_ 20 % Gd, $*p* < 0.05 compared to Gd_2_O_3_

While all three CeO_2_ nanoparticles did not generate hydroxyl radicals in an acellular system, previous studies have shown that CeO_2_ induces significant ROS in vitro [6, 7]; thus, cellular EPR was completed. The results showed that in RLE-6TN cells, all three CeO_2_ nanoparticles significantly reduced the presence of hydroxyl radicals; however, in NR8383 cells, only pure CeO_2_ and CeO_2_ 10 % Gd significantly scavenged the free radicals. In both cell lines, the Gd_2_O_3_ control had no effect (Figs. 6 and 7).

### CeO_2_ Nanoparticle Exposure Effects on Cell Viability

**Fig. 8:**
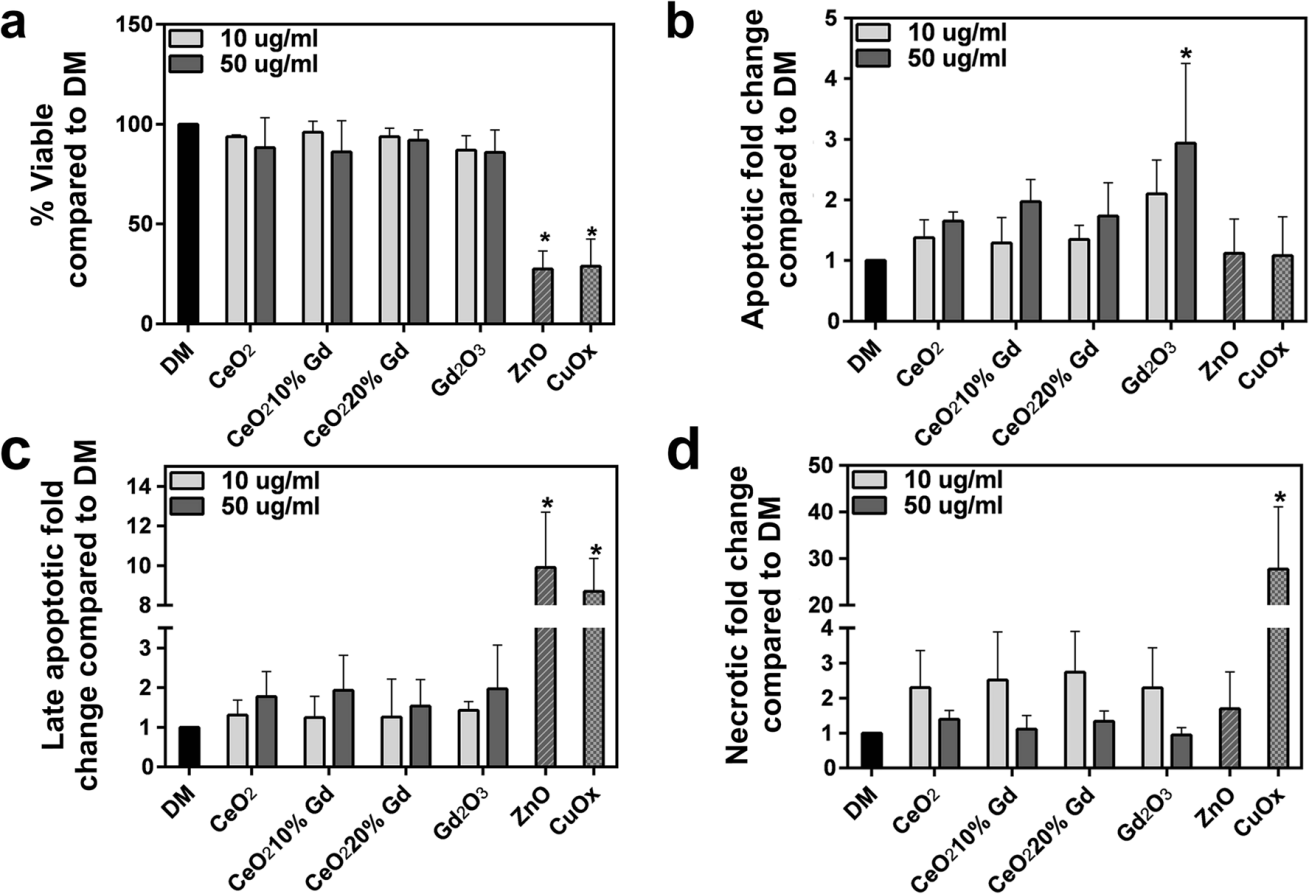
CeO_2_ nanoparticles cause no significant changes in RLE-6TN cell viability at 24 h. **a** RLE-6TN cells were exposed to CeO_2_ at 10 or 50 μg/ml for 24 h (Gd_2_O_3_ at 1.79 or 8.95 μg/ml). Collected cells were incubated with annexin V/propidium iodide on ice for 15 min then run, and 10,000 events were measured. Graph represents cells that were viable after 24 h. ZnO and CuOx, at 50 μg/ml, were used as positive controls for apoptosis and necrosis, respectively, and DM was used as a negative control. *Error bars* represent mean ± standard deviation. *p < 0.05 compared to control. **b** Cells stained positive for annexin V. **c** Cells stained positive for both annexin V and propidium iodide. **d** Cells stained positive for propidium iodide

To measure CeO_2_ effects on apoptosis and necrosis at 24 h, an annexin V/propidium iodide dual stain was used. At 24 h, no CeO_2_ nanoparticle affected overall cell viability in RLE-6TN cells at either 10 or 50 μg/ml doses. Gd_2_O_3_ induced significant apoptosis (annexin V positive) at 8.95 μg/ml compared to the control (Fig. 8).

**Fig. 9:**
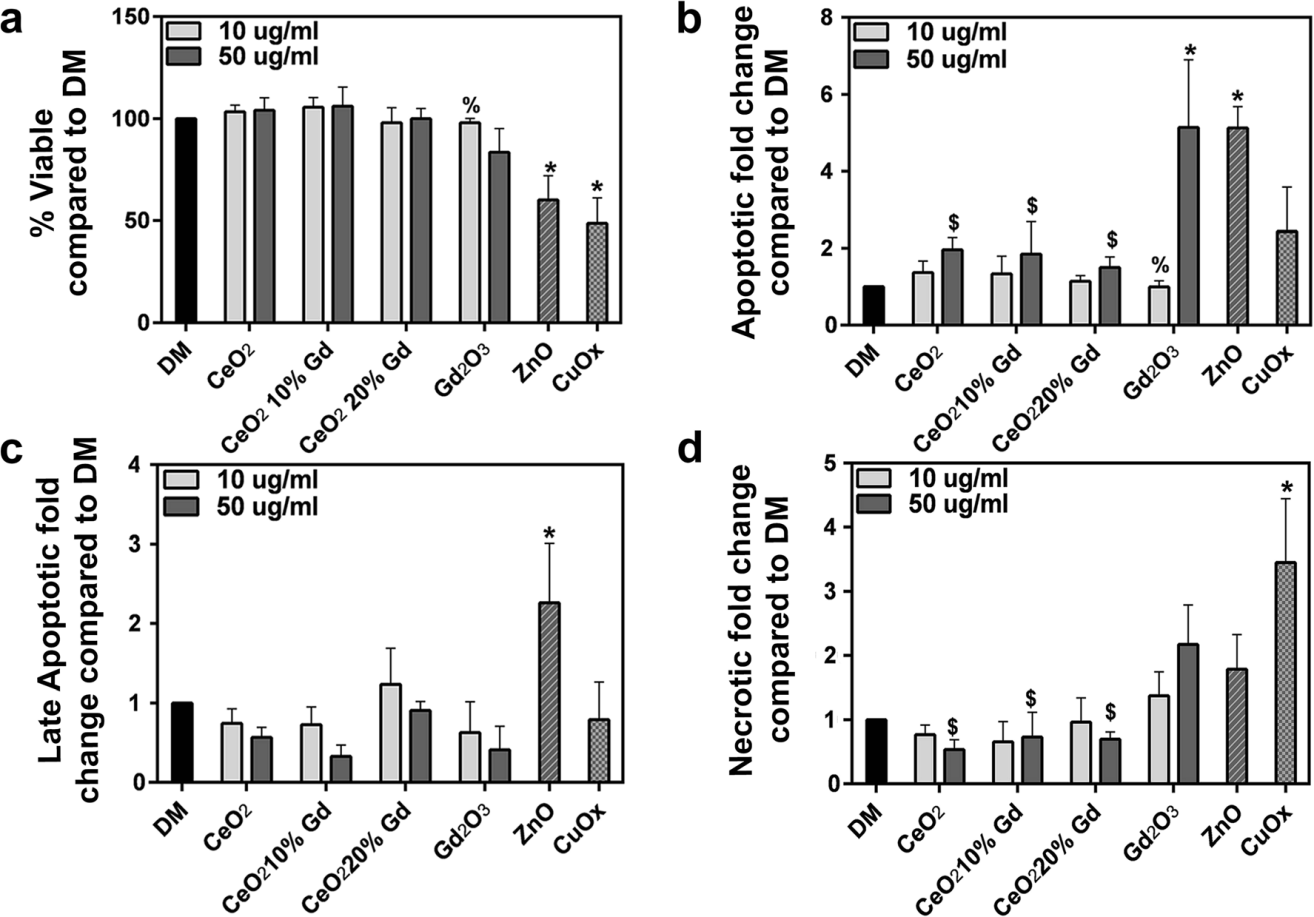
CeO_2_ nanoparticles cause no significant changes in NR8383 cell viability at 24 h. **a** NR8383 cells were exposed to CeO_2_ at 10 or 50 μg/ml for 24 h (Gd_2_O_3_ at 1.79 or 8.95 μg/ml). Collected cells were incubated with annexin V/propidium iodide on ice for 15 min then run, and 10,000 events were measured. Graph represents cells that were viable after 24 h. ZnO and CuOx, at 50 μg/ml, were used as positive controls for apoptosis and necrosis, respectively, and DM was used as a negative control. *Error bars* represent mean ± standard deviation. *p < 0.05 compared to control, %*p* < 0.05 compared to 50 μg/ml, $*p* < 0.05 compared to Gd_2_O_3_ at equivalent dose. **b** Cells stained positive for annexin V. **c** Cells stained positive for both annexin V and propidium iodide. **d** Cells stained positive for propidium iodide

In NR8383 cells, pure CeO_2_, doped CeO_2_, and Gd_2_O_3_ nanoparticles had no significant effects on overall cell viability or development of necrosis. However, Gd_2_O_3_ significantly increased the number of cells undergoing apoptosis at a dose of 8.95 μg/ml compared to the control (Fig. 9).

## Discussion

As industrial interest in the use of CeO_2_ nanoparticles increases so will manufacturing and worker exposures. While disagreements exist within the literature as to the nature of CeO_2_ toxicity, it is almost universally agreed upon that CeO_2_ affects ROS, theoretically due to its exceptional redox potential. Therefore, this study focused on examining how altering the valence state of CeO_2_ nanoparticles through doping affects CeO_2_ toxicity, specifically its effects on ROS generation.

As predicted, the use of Gd_2_O_3_ as a dopant substantially altered the Ce^3+^ to Ce^4+^ ratio of the nanoparticles (Table 1) [4]. XPS analysis of powder surfaces indicated that doping with Gd_2_O_3_ increased the rate of reduction of Ce^4+^ to Ce^3+^, a rate that increased as the concentration of Gd_2_O_3_ increased. CeO_2_ containing 10 mol% Gd_2_O_3_-doped into the nanoparticles had a ratio shift from 16 to 42 % compared to pure CeO_2_, while the 20 mol% Gd_2_O_3_-doped nanoparticles shifted the ratio from 16 to 44 %. Alternatively, a study completed by Celado et al. showed that doping with samarium (Sm) decreased the amount of Ce^3+^ in the nanoparticles [9]. This difference in doping outcome may be a result of Gd_2_O_3_ to introduce more Ce^3+^ oxidation state into the nanoparticle compared to Sm as previously shown [23]. While the effects of doping observed in the two studies conflict, our results correlate with the general finding that as doping increases, antioxidant potential decreases. Thus, in conjunction with the works of Celado et al., it appears that the ratio of Ce^3+^/Ce^4+^ is not as crucial in determining antioxidant potential of CeO_2_ nanoparticles as is the ability of Ce to transition between the two valence states. This transitional ability is hindered following doping since the Ce nanoparticles are forced toward one valence state and, due to the stability of Gd in the lattice structure, unable to transition as easily to the other state [4]. Further support of this effect is the mere change in 3+/4+ ratio between the 10 mol% Gd_2_O_3_ and 20 mol% Gd_2_O_3_-doped CeO_2_ nanoparticles from 42 to 44 % (Table 1) accompanied by the dramatic change in antioxidant potential of the two nanoparticles. This decreased antioxidant effect was most notable in the cellular EPR model, where CeO_2_ 20 % Gd was significantly different in its scavenging abilities when compared to the pure CeO_2_ and CeO_2_ 10 % Gd (Figs. 6 and 7). Thus, it appears that the valence state of CeO_2_ is less important in determining antioxidant ability than the capacity of CeO_2_ to transition between the two valence states. Differences in scavenging ability also existed between the two cell lines; specifically, CeO_2_ 20 % Gd had no significant effect on induced free radicals in NR8383 cells, whereas it was able to significantly reduce hydroxyl radical formation in RLE-6TN cells. While this was unexpected, discrepancies between cell lines are not unusual, especially in CeO_2_ nanoparticle toxicity studies, and may be the result of differences in cellular physiology and function [6, 24]. Thus, in these studies, it appears that CeO_2_ is a less efficient antioxidant in NR8383 cells and that doping has a more pronounced effect on responses of macrophages than those of epithelial cells. The Gd_2_O_3_ had no significant effects on ROS in either EPR model, implying that the antioxidant abilities of the CeO_2_ are due to the presence of Ce^3+^, Ce^4+^, or oxygen vacancies, and not the dopant.

To ensure that differences in cellular-reactivity were not due to differences in association between the particles and the cells, enhanced dark field microscopy was utilized. All of the nanoparticles were capable of associating with both cell types in a matter of minutes (Fig. 3), suggesting that the cells would be capable of responding in the short time course conducted in EPR studies and, further, that measured EPR differences were not due to differences in cellular association. These results were anticipated based on zeta-potential (Table 2) and imply that the presence of Gd did not alter important surface chemistry necessary for interaction of CeO_2_ with cells. Increased concentrations of Gd also did not alter the observed size of the nanoparticles (data not shown), implying that differences in reactivity are not a result of differences in size. The hydrodynamic diameters of the CeO_2_ 10 % Gd and CeO_2_ 20 % Gd particles in DM were smaller than the pure CeO_2_; this difference in hydrodynamic size compared to measured size from SEM is attributed to the sonication of the particle suspensions prior to DLS measurement. Agglomeration is central in nanoparticle-cellular interactions and reactivity [25] and may therefore be important in describing differences in antioxidant potential; however, if agglomeration was important in describing these results, it would also be expected that differences in toxicity between the nanoparticles would be measured. No cytotoxicity was measured in this study; thus, this lack of correlation suggests that the differences in antioxidant abilities are due to valence state and transitional ability rather than variances in nanoparticle agglomeration.

None of the three CeO_2_ nanoparticles induced significant changes in overall cell viability and did not induce apoptosis or necrosis at 24 h (Figs. 8 and 9). While the lack of differences between the CeO_2_ nanoparticles was unexpected, numerous studies have shown a lack of CeO_2_ reactivity at similar doses [9, 26] and have accounted this nontoxic effect to CeO_2_ transitional ability and presence of Ce^3+/4+^. In agreement, Celardo et al. [9] also reported that doping had no effect on cellular viability, again implying that changes in viability measured in other CeO_2_ nanoparticle studies are not likely a result of valence state.

To further elucidate the effect of Gd_2_O_3_ on differences in CeO_2_ toxicity, annexin V/PI dual staining was completed and implied that at a concentration equivalent to the quantity of Gd_2_O_3_ in the 50 μg/ml dose of CeO_2_ 20 % Gd, the pure Gd_2_O_3_ caused significant apoptosis at 24 h in both cell lines (Figs. 8 and 9). In fact, all three CeO_2_ nanoparticles did not elicit apoptosis. This implies that Gd did not separate from the doped nanoparticles and interact with the cells to yield the observed effects.

Previous studies have suggested that the valence state of Ce in CeO_2_ nanoparticles is important in toxicity and ROS production [3, 10]; however, attempts to elucidate which valence state is important for biological effects are lacking. This study attempted to confirm, through alterations in CeO_2_ valence state ratio, that a specific valence state is a less important determinant of CeO_2_ reactivity than the presence of mixed valence state and transitional ability. Overall, our initial findings suggest that doping does not increase toxicity but appears to inhibit CeO_2_ antioxidant potential in a rapid cellular exposure in support of our hypothesis. Since CeO_2_ toxicity results greatly differ between in vitro and in vivo models [10, 27–29], further studies will need to be completed to determine the effect of valence state on toxicity in vivo.

## Electronic supplementary material

Below is the link to the electronic supplementary material.

ESM 1 (PDF 209 kb)

ESM 2 (PDF 251 kb)
