# Supplementary material for: The Effect of Cerium Oxide Nanoparticle Valence State on Reactive Oxygen Species and Toxicity
Source: Biol Trace Elem Res. 2015 Mar 17;166(1):96–107. doi: 10.1007/s12011-015-0297-4 (PMC4469090; doi:10.1007/s12011-015-0297-4)
Supplement: Supplementary file 2 — (PDF 251 kb) [file 12011_2015_297_MOESM2_ESM.pdf]

Title: The Effect of Cerium Oxide Nanoparticle Valence State on Reactive Oxygen Species and Toxicity

Biological Trace Elements Research

Authors: Katherine M Dunnick[*†], Rajalekshmi Pillai[‡], Kelly L Pisane[§¥], Aleksandr B Stefaniak[¥], Edward M Sabolsky[‡], Stephen S Leonard[*†]

[*]National Institute for Occupational Safety and Health, HELD, Morgantown, WV 26505
[†]West Virginia University, Pharmaceutical and Pharmacological Sciences, Morgantown, WV 26505
[‡]WVU Benjamin M. Statler College of Engineering and Mineral Resources, Morgantown, WV 26505
[§]West Virginia University, Department of Physics and Astronomy, Morgantown, WV 26505
[¥]National Institute for Occupational Safety and Health, DRDS, Morgantown, WV 26505

Corresponding Author: Katherine Dunnick
E-mail: kdunnick@mix.wvu.edu

Online Resource 2

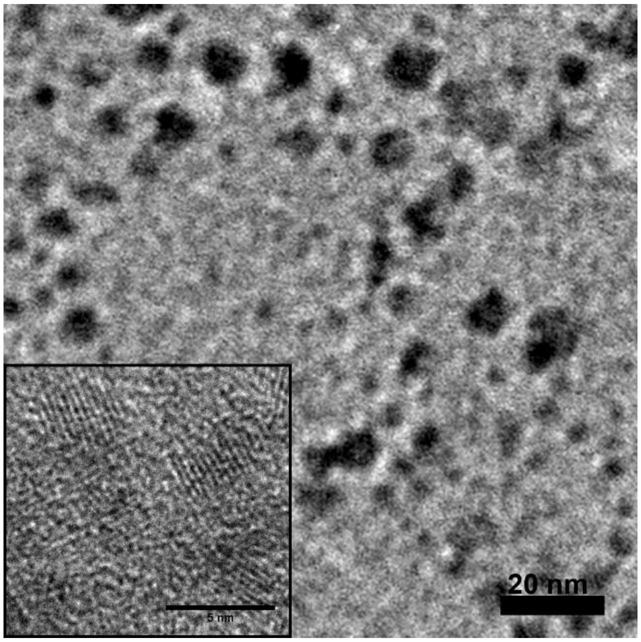

Online Resource 2 TEM images of pure CeO_2 nanoparticles. Scale bar, 20 nm, inlay 5 nm
